# The Effects of Multiple Maillard Reaction Products in Infant Formula on Host Immunity and Neurotoxicity

**DOI:** 10.3390/foods15142533

**Published:** 2026-07-17

**Authors:** Qiaosi Wei, Lili Jia, Xiangxin Wang, Shubo Luo, Dongying Cui, Jufang Li, Qinggang Xie, Yajun Xu

**Affiliations:** 1 Department of Nutrition and Food Hygiene, School of Public Health, Peking University, No. 38 Xueyuan Road, Haidian District, Beijing 100083, China; 2 PKUHSC-China Feihe Joint Research Institute of Nutrition and Healthy Lifespan Development, Xueyuan Road 38, Haidian, Beijing 100083, China; 3 Beijing Key Laboratory of Toxicological Research and Risk Assessment for Food Safety, Peking University, No. 38 Xueyuan Road, Beijing 100083, China

**Keywords:** Maillard reaction products, infant formula, zebrafish, immunotoxicity, neurotoxicity

## Abstract

Various Maillard reaction products (MRPs) are formed in infant formula during thermal processing and storage. However, safety assessments are often based on individual compounds, which may not adequately reflect the potential risks associated with exposure to multiple coexisting components in real food products. Therefore, this study used GC–MS to screen for typical MRPs in infant formula. Based on the detection results, glyoxal, 2-acetylfuran, 2-furfural, and 5-hydroxymethylfurfural (5-HMF) were selected to establish a mixed-exposure system. Subsequently, using zebrafish as a model, we systematically evaluated the toxic effects of two mixture concentrations, 1/9 maximum non-lethal concentration (MNLC) and 1/3 MNLC, on the immune system, nervous system, gastrointestinal tract, and liver. The results showed that these four MRPs coexisted in infant formula and that combined exposure induced significant biological damage even at low doses. Both mixture concentrations significantly reduced neutrophil and macrophage levels and promoted apoptosis in central nervous system cells. The higher-concentration mixture further reduced T-cell counts, suppressed motor neuron development, and decreased locomotor activity during the light phase. In addition, the higher-concentration mixture decreased the gastrointestinal area, increased the liver area, delayed yolk sac absorption, and caused marked histopathological damage. Compared with single-compound exposure, combined exposure exerted more pronounced effects on immune- and neuro-related endpoints, suggesting synergistic interactions among different MRPs and identifying the immune and nervous systems as the more sensitive targets of toxicity. In summary, mixed exposure to typical MRPs in infant formula may pose a greater biological risk than that predicted by evaluations based on individual components alone. This study provides experimental evidence for identifying combined toxicity and improving the safety risk assessment of MRPs in thermally processed infant foods.

## 1. Introduction

The Maillard reaction is one of the most common non-enzymatic browning reactions that occur during food processing and storage. It involves reactions between the carbonyl groups of reducing sugars and the amino groups of proteins, peptides, or free amino acids, leading to the formation of a series of intermediates and end products, such as Amadori products, α-diketones, furan compounds, and advanced glycation end products (AGEs) [1]. Although this reaction contributes to the color, aroma, and flavor of food [2], it may also reduce nutritional quality and generate potentially harmful low-molecular-weight products [3]. Therefore, its dual implications for food quality and safety have long been a major focus in food chemistry and food safety research. In dairy products, cereal-based products, and infant foods, Maillard reactions and their products warrant particular attention because of the abundance of reactive substrates and the frequent use of thermal processing [4]. Infant formula is a dairy product rich in protein, fat, and lactose. In addition, infant formula is commonly produced by spray drying. These characteristics may promote Maillard reactions and lead to the accumulation of various Maillard reaction products (MRPs). Previous studies have shown that multiple thermal reaction markers are formed in infant formula during processing and storage, including Amadori compounds, AGEs, α-diketones, and furan compounds [5,6,7]. Furthermore, heat treatment affects lysine availability and protein digestibility, suggesting that Maillard reactions not only alter product quality but may also influence biological effects and potential health risks [8]. Among these MRPs, glyoxal, 2-furfural, 2-acetylfuran, and 5-hydroxymethylfurfural are particularly representative. Glyoxal is a highly reactive α-dicarbonyl compound and a key precursor of AGEs, which can trigger protein modification, carbonyl stress, and oxidative damage [9]. 5-HMF and furfural derivatives are dehydration and pyrolysis products that are widely generated during thermal food processing [10]. Reports indicate that excessive exposure to 5-HMF is associated with host oxidative stress, developmental toxicity, and organ-specific damage, and its toxicity may be related to metabolic transformation within the body [11,12]. 2-Furfural primarily targets the liver in animal studies, inducing hepatocellular vacuolization, inflammation, and tumor-related changes after repeated exposure [13]. Furthermore, 2-Acetylfuran has been associated with genotoxicity alerts because furan-related flavoring substances can form reactive metabolites capable of damaging DNA [14].

Recent studies on infant formula have further indicated that MRPs do not occur as isolated compounds, but coexist within the same processing system and final product, where they continue to undergo transformation and accumulation during storage [5,15]. Although numerous studies have examined the formation mechanisms, analytical methods, and specific toxic effects of individual MRPs, consumers are more likely to experience simultaneous exposure to multiple MRPs under real-world dietary conditions. Combined exposure has become an important issue in modern food and environmental toxicology. Under conditions of long-term, low-dose exposure, mixture effects cannot be reliably extrapolated from results obtained with individual compounds [16]. Therefore, establishing representative MRPs mixture systems based on actual food exposure characteristics and evaluating their multi-organ, multi-endpoint toxicity are of practical significance for improving the safety assessment of infant formula [17]. Infants and young children are in a stage of rapid growth and development, and their immune, nervous, and digestive/metabolic systems are not yet fully mature. As a result, they are generally more sensitive to exogenous chemical exposure. Existing research suggests that thermal processing byproducts and reactive carbonyl compounds can disrupt physiological homeostasis through mechanisms including oxidative stress, protein adduct formation, apoptosis, and metabolic dysfunction [6,18].

Owing to its transparent embryos, rapid development, well-defined genetic background, amenability to visualizing immune and neural development, and suitability for simultaneous multi-organ toxicity assessment, zebrafish has become an important model organism for screening food chemicals and developmental toxicants [19,20]. In particular, transgenic zebrafish enable in situ observation of changes in neutrophils, macrophages, T cells, motor neurons, and organ morphology. In addition, endpoints such as light-dark behavior, neuronal apoptosis, and gastrointestinal and hepatic morphology are widely used in toxicological phenotypic analyses. Therefore, the zebrafish model provides an efficient and integrated experimental platform for investigating immune, neurological, gastrointestinal, and hepatic toxicity induced by combined exposure to MRPs. In this study, GC–MS was used to screen for major MRPs in infant formula. Based on these results and previous single-compound toxicity data, four representative MRPs detected in infant formula—glyoxal, 2-acetylfuran, 2-furfural, and 5-HMF—were selected to establish a mixed-exposure system. The zebrafish model was then used to systematically evaluate the effects of this mixture on the immune system, nervous system, gastrointestinal tract, and liver. Furthermore, the toxicity of the mixture was compared with that of individual compounds to determine whether synergistic effects exist among typical MRPs in infant formula.

## 2. Materials and Methods

### 2.1. Chemicals and Reagents

The key chemicals and their sources were as follows: methylcellulose (Lot No. C2004046, Shanghai Aladdin Biochemical Technology Co., Ltd., Shanghai, China), dimethyl sulfoxide (DMSO; Lot No. BCCD8942, Sigma, St. Louis, MO, USA), acridine orange (AO; Lot No. C12894919, Shanghai Maclin Biochemical Technology Co., Ltd., Shanghai, China), 4% tissue fixative (Lot No. 20230401, Beijing Solarbio Technology Co., Ltd., Beijing, China), xylene (Lot No. 20230424, Sinopharm Chemical Reagent Co., Ltd., Shanghai, China), Mayer’s hematoxylin staining solution (Lot No. 20220120, Shanghai Yihe Biotechnology Co., Ltd., Shanghai, China), eosin staining solution (Lot No. 20220120, Shanghai Yihe Biotechnology Co., Ltd., Shanghai, China), neutral gum (Lot No. 330A021, Beijing Solarbio Technology Co., Ltd., Beijing, China), and paraffin for sectioning (Lot No. 20230322, Shanghai Huayong Paraffin Co., Ltd., Shanghai, China). All other chemicals and reagents used in this study were of analytical or HPLC grade.

### 2.2. Quantification of Glyoxal in Formula Powder by GC–MS

#### 2.2.1. Preparation of Reference Standards

Twelve reference standards were prepared by dissolving 0.100 ± 0.005 g of each compound in 200 mL of methanol to obtain stock solutions at a concentration of 0.5 mg/mL.

#### 2.2.2. Sample Preparation

Three common infant formulas were selected for the determination of twelve Maillard reaction products. The three infant formulas are commercially available products. Their main ingredients include cow’s milk, demineralized whey powder, lactose, vegetable oil, OPO structured fats, vitamins, and minerals. They are prepared using a spray-drying method, with heat treatment involved in the sterilization, concentration, and spray-drying steps. The spray-drying process involves high-temperature exposure at 160–180 °C. Ten grams of each formula powder sample was dissolved in 200 mL of methanol. The solution was mixed thoroughly, and 1 mL was transferred to a GC–MS vial for injection using the autosampler. One milliliter of each standard solution was transferred to a sample vial, and the retention time and peak area were determined by GC–MS. The twelve Maillard reaction products in the samples were qualitatively identified by comparison with the chromatographic peaks of the standard solutions.

#### 2.2.3. Qualitative Analysis by GC–MS

Qualitative analysis was performed using a gas chromatography–mass spectrometry (GC–MS) system equipped with an HP-5 column (Agilent Technologies, Santa Clara, CA, USA.) (30 m × 0.32 mm, 0.25 μm). The GC–MS conditions were as follows: flow rate, 10 mL/min; column temperature, 80–230 °C; injection volume, 10 µL; and temperature ramp rate, 10 °C/min. Data were subsequently acquired and processed using GC–MS analysis software (Masshunter B.08.00). Each formula powder sample was analyzed in triplicate.

### 2.3. Preparation of Maillard Reaction Product Mixtures

The Maillard reaction products included in the follow-up study were those detected in at least two infant formulas (Table 1). Four Maillard reaction products were identified, and mixture groups for the follow-up study were established based on their concentrations. The mixtures consisted of glyoxal, 2-acetylfuran, 2-furfural, and 5-hydroxymethylfurfural, for which dose–response relationships had been reported previously [21,22]. In this study, 1/9 of the maximum non-lethal concentration (MNLC) of each compound was defined as Complex 1, and 1/3 of the MNLC was defined as Complex 2. Specifically, Complex 1 contained 0.263 μL/mL glyoxal, 0.094 μL/mL 2-acetylfuran, 0.001 μg/mL 2-furfural, and 16.900 μg/mL 5-HMF. Complex 2 contained 0.790 μL/mL glyoxal, 0.281 μL/mL 2-acetylfuran, 0.003 μg/mL 2-furfural, and 274.000 μg/mL 5-HMF. The mixtures administered to zebrafish were dissolved in 1% DMSO. In addition to the Complex 1 and Complex 2 groups, a normal control (NC) group and a solvent control (DMSO) group were included. Animal use was approved by the Institutional Animal Care and Use Committee (IACUC-2023-6809-01). All procedures complied with the institutional license for experimental animals (SYXK (Zhejiang) 2022-0004) and adhered to AAALAC accreditation requirements (accreditation No. 001458).

### 2.4. Fluorescence Analysis of Neutrophils

A total of 120 transgenic zebrafish with green fluorescent neutrophils at 3 days post-fertilization were randomly divided into four groups of 30 fish each. Each group was housed in a six-well plate containing 3 mL of water per well. The test compounds were added directly to the rearing water. After 2 days of exposure at 28 °C, 10 zebrafish were randomly selected from each group and imaged under a fluorescence microscope. Images were analyzed using NIS-Elements D 3.20 software to determine the number of neutrophils in the caudal vein.

### 2.5. Fluorescence Analysis of T Cells

A total of 120 transgenic zebrafish expressing red fluorescent T cells at 3 days post-fertilization were randomly divided into four groups. Grouping and exposure procedures were the same as those described in Section 2.4. After 2 days of exposure at 28 °C, 10 zebrafish were randomly selected from each group and imaged under a fluorescence microscope. Images were analyzed using NIS-Elements D 3.20 software to assess T-cell fluorescence intensity.

### 2.6. Fluorescence Analysis of Macrophages

A total of 120 transgenic zebrafish with green fluorescent macrophages at 3 days post-fertilization were randomly divided into four groups. Grouping and exposure procedures were the same as those described in Section 2.4. After 2 days of exposure at 28 °C, 10 zebrafish were randomly selected from each group and imaged under a fluorescence microscope. Images were analyzed using NIS-Elements D 3.20 software to assess macrophage fluorescence intensity in the caudal vein.

### 2.7. Analysis of Zebrafish Locomotion

A total of 120 wild-type AB zebrafish at 4 days post-fertilization were randomly divided into four groups. Grouping and exposure procedures were the same as those described in Section 2.4. After 1 day of exposure at 28 °C, 10 zebrafish were randomly selected from each group and placed in a behavioral analyzer to measure total swimming distance in the dark for 20 min.

### 2.8. Analysis of Apoptotic Cells in the Zebrafish Central Nervous System

A total of 120 wild-type AB zebrafish at 6 h post-fertilization were randomly divided into four groups. Grouping and exposure procedures were the same as those described in Section 2.4. After 2 days of exposure at 28 °C, zebrafish in each group were stained with AO in the dark for 30 min. After three washes with standard diluent, 10 zebrafish were randomly selected from each group and imaged under a fluorescence microscope. Images were analyzed using NIS-Elements D 3.20 software to quantify the fluorescence intensity of apoptotic cells in the central nervous system.

### 2.9. Analysis of Peripheral Motor Neuron Length in Zebrafish

A total of 120 transgenic zebrafish expressing green fluorescent motor neurons at 6 h post-fertilization were randomly divided into four groups. Grouping and exposure procedures were the same as those described in Section 2.4. After 3 days of exposure at 28 °C, 10 zebrafish were randomly selected from each group and imaged under a fluorescence microscope. Images were analyzed using NIS-Elements D 3.20 software to measure peripheral motor neuron length in the region spanning the three somites above the cloacal opening.

### 2.10. Analysis of Gastrointestinal Area

A total of 120 wild-type AB zebrafish at 3 days post-fertilization were randomly divided into four groups. After 2 days of exposure at 28 °C, 10 zebrafish were randomly selected from each group and imaged under a dissecting microscope. Images were analyzed using NIS-Elements D 3.20 software to determine gastrointestinal area.

### 2.11. Analysis of Neutrophil Counts in the Gastrointestinal Tract

A total of 120 transgenic zebrafish expressing green fluorescent neutrophils at 3 days post-fertilization were randomly divided into four groups. Grouping and exposure procedures were the same as those described in Section 2.4. After 2 days of exposure at 28 °C, 10 zebrafish were randomly selected from each group and imaged under a fluorescence microscope. Images were analyzed using NIS-Elements D 3.20 software to determine the number of gastrointestinal neutrophils per unit area.

### 2.12. Liver Morphological Analysis

A total of 120 wild-type AB zebrafish at 3 days post-fertilization were randomly divided into four groups. Grouping and exposure procedures were the same as those described in Section 2.4. After 2 days of exposure at 28 °C, 10 zebrafish were randomly selected from each group and imaged under a dissecting microscope. Images were analyzed using NIS-Elements D 3.20 software to assess liver area, mean liver brightness, and the area of delayed yolk sac absorption.

### 2.13. H&E Analysis of Liver Tissue

A total of 120 wild-type AB zebrafish at 3 days post-fertilization were randomly divided into four groups. Grouping and exposure procedures were the same as those described in Section 2.4. After 2 days of exposure at 28 °C, zebrafish were fixed in 4% tissue fixative. The fixed samples underwent standard histological processing, including graded ethanol dehydration, clearing, paraffin embedding, and serial sectioning, followed by hematoxylin and eosin (H&E) staining. Finally, morphological changes and histopathological damage in zebrafish liver tissue were examined under a light microscope.

### 2.14. Statistical Analysis

Data are presented as the mean ± standard deviation, with a sample size of 10 per group. Comparisons between two groups were performed using unpaired t-tests. Statistical significance was defined as * *p* < 0.05, ** *p* < 0.01, and *** *p* < 0.001, whereas non-significant differences were denoted as “ns.”

## 3. Results

### 3.1. Identification of Maillard Reaction Products in Infant Formula

The Maillard reaction is a common chemical reaction in foods that generates a variety of intermediate and end products. Infant formula is rich in proteins and carbohydrates, which provide abundant substrates for the Maillard reaction. In addition, infant formula is commonly produced by spray drying, and the high temperatures involved in this process further promote the Maillard reaction. Therefore, 12 MRPs were selected, and their retention times and peak characteristics were identified by GC–MS. As shown in Table 1, the retention times of the 12 MRPs ranged from 2.110 to 14.372 min. Subsequently, these 12 MRPs were analyzed in three infant formula samples. The results showed that 2-acetylfuran, 2-furfural, and 5-hydroxymethylfurfural were detected in all infant formula samples. In addition, glyoxal was detected in two infant formula samples. The remaining eight Maillard reaction products were below the limit of detection in all infant formula samples. Overall, these results indicate that multiple categories of Maillard reaction products are present in infant formula, with 2-acetylfuran, 2-furfural, 5-hydroxymethylfurfural, and glyoxal being the predominant compounds detected.

In our previous study, we analyzed the dose–response relationships of 2-acetylfuran, 2-furfural, 5-hydroxymethylfurfural, and glyoxal with respect to zebrafish toxicity. The results showed that none of the four compounds exhibited detectable toxicity at 1/9 MNLC. At 1/3 MNLC, 2-acetylfuran and 5-hydroxymethylfurfural exhibited mild toxicity. Notably, Maillard reaction products in infant formula and other foods do not occur in isolation; multiple compounds are often generated simultaneously. Therefore, the toxic dose of a single compound may not fully reflect the risks associated with combined exposure to Maillard reaction products. In this study, we evaluated the toxicity of the four-compound mixture at concentrations corresponding to 1/9 MNLC and 1/3 MNLC.

### 3.2. Effects of the Mixtures on Zebrafish Immunity

**Figure 1:**
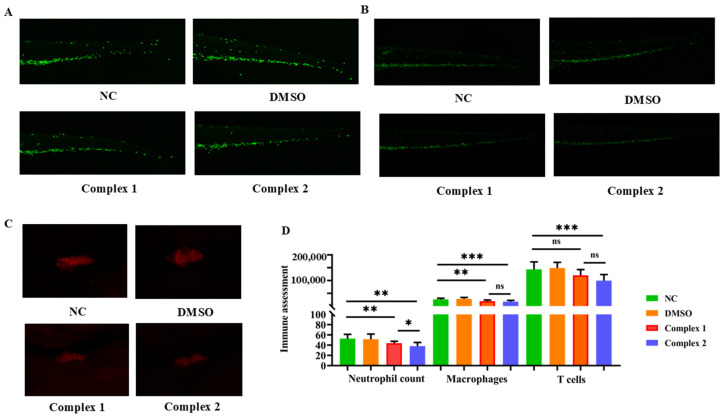
Immunotoxicity of Maillard reaction product complexes. (**A**) Neutrophils, (**B**) Macrophages, (**C**) T cells. (**D**) Quantitative fluorescence analysis of neutrophils, macrophages, and T cells. * *p* < 0.05, ** *p* < 0.01, *** *p* < 0.001, ns indicates no significant difference. n = 10.

The effects of the mixtures on three types of immune cells were evaluated using transgenic zebrafish models in which neutrophils and macrophages were labeled with green fluorescence and T cells with red fluorescence. Representative fluorescence images are shown in Figure 1A–C. Compared with the NC group, neutrophil fluorescence intensity was significantly reduced in both the Complex 1 and Complex 2 groups (*p* < 0.01, Figure 1A), indicating neutrophil toxicity at both mixture concentrations (Figure 1D). Furthermore, neutrophil fluorescence intensity was significantly lower in the Complex 2 group than in the Complex 1 group (*p* < 0.01, Figure 1B). This finding indicates that neutrophil toxicity increased with mixture concentration. Compared with the NC group, macrophage fluorescence intensity was significantly reduced in both the Complex 1 and Complex 2 groups (*p* < 0.01, Figure 1C), whereas no significant difference was observed between the two mixture groups (*p* > 0.05). In addition, T-cell fluorescence intensity was significantly lower in the Complex 2 group than in the NC group, whereas no significant difference was observed between the Complex 1 and NC groups. These results indicate that Complex 2 exerted greater immunotoxicity than Complex 1.

### 3.3. Effects of the Mixtures on the Zebrafish Nervous System

**Figure 2:**
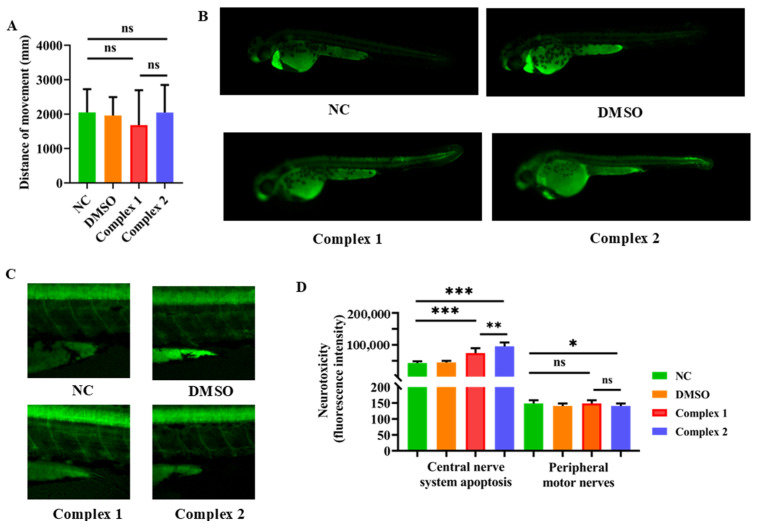
Neurotoxicity of Maillard reaction product complexes. (**A**) Zebrafish movement distance in the dark for 20 min. (**B**) Central nervous system cell apoptosis. (**C**) Motor nerve length. (**D**) Quantitative fluorescence analysis of central nervous system cell apoptosis and motor nerve length. * *p* < 0.05, ** *p* < 0.01, *** *p* < 0.001, ns indicates no significant difference. n = 10.

Zebrafish locomotor activity is an external manifestation of nervous system function. Changes in zebrafish locomotor activity under light and dark conditions are associated with behavioral patterns, sensory function, and physiological responses. During the initial 5 min light phase, locomotor speed was significantly reduced in both the Complex 1 and Complex 2 groups compared with the NC group (*p* < 0.01, Table 2). During the second light phase (11–15 min), swimming speed in the Complex 1 group was significantly lower than that in the NC group (*p* < 0.05). Notably, no significant differences in swimming speed were observed among the groups during either dark phase (6–10 min and 16–20 min; *p* > 0.05). Consistent with these findings, no significant differences in total swimming distance were observed among the four groups during the 20 min dark period (*p* > 0.05, Figure 2A). We next analyzed apoptosis in the central nervous system and peripheral motor neuron length (Figure 2B,C). Compared with the NC group, apoptosis in the central nervous system was significantly increased in both the Complex 1 and Complex 2 groups (*p* < 0.001, Figure 2A), with significantly higher levels in the Complex 2 group than in the Complex 1 group (*p* < 0.01). In addition, peripheral motor neuron length was significantly reduced in the Complex 2 group compared with the NC group, whereas no significant difference was observed between the Complex 1 and NC groups (*p* < 0.05, Figure 2B). These results indicate that the MRP mixtures induced neurotoxicity and impaired zebrafish locomotor activity under light conditions. Overall, Complex 2 exerted greater neurotoxic effects than Complex 1.

### 3.4. Effects of the Mixtures on the Zebrafish Gastrointestinal Tract

**Figure 3:**
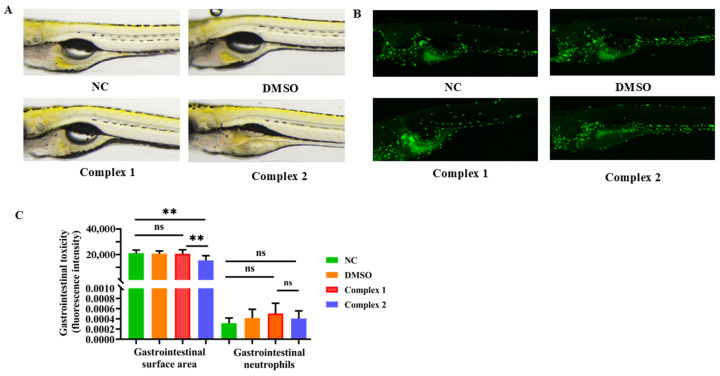
Gastrointestinal toxicity of Maillard reaction product complexes. (**A**) Visual image of the gastrointestinal tract. (**B**) Number of neutrophils in the gastrointestinal tract. (**C**) Quantification of gastrointestinal tract area and number of neutrophils per unit area. ** *p* < 0.01, ns indicates no significant difference. n = 10.

The effects of the MRP mixtures on gastrointestinal morphology were evaluated in wild-type zebrafish by measuring changes in gastrointestinal area (Figure 3A). No significant difference in gastrointestinal area was observed between the Complex 1 and NC groups (*p* > 0.05, Figure 3C). However, when the mixture concentration was increased to 1/3 MNLC, gastrointestinal area decreased significantly (*p* < 0.01). In particular, gastrointestinal area in the Complex 2 group was significantly lower than that in the Complex 1 group (*p* < 0.01). The number of neutrophils in the zebrafish gastrointestinal tract was then analyzed (Figure 3B). Gastrointestinal neutrophils contribute to microbiota-associated immune homeostasis and host defense against pathogens [23,24]. No significant differences in gastrointestinal neutrophil counts were observed among the four groups (*p* > 0.05, Figure 3C). These results suggest that the MRP mixtures primarily affected gastrointestinal morphology rather than inducing detectable immune alterations within the gastrointestinal tract.

### 3.5. Effects of the Mixtures on the Zebrafish Liver

**Figure 4:**
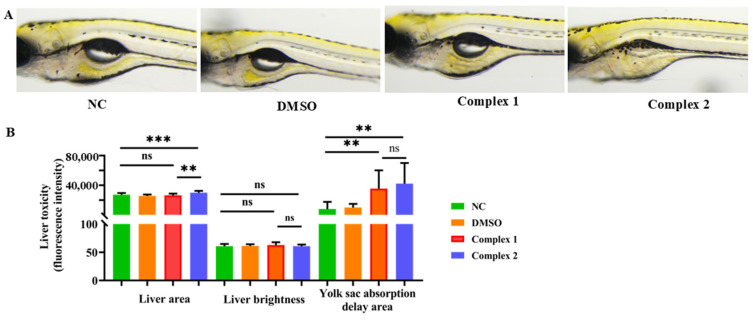
Hepatotoxicity of Maillard reaction product complexes. (**A**) Visual images of two livers. (**B**) Liver area, liver brightness, and area of delayed yolk sac absorption. ** *p* < 0.01, *** *p* < 0.001, ns indicates no significant difference. n = 10.

The liver is the primary organ responsible for detoxification and biotransformation in zebrafish and other vertebrates. Toxic substances absorbed from the intestine enter the circulation, reach the liver, and are metabolized or eliminated by hepatic enzymes. Accordingly, liver function plays a critical role in limiting toxicity induced by Maillard reaction products. Therefore, the effects of the MRP mixtures on liver area, liver brightness, and delayed yolk sac absorption area were analyzed to assess hepatotoxicity (Figure 4A,B). Compared with the NC group, the delayed yolk sac absorption area was significantly increased in the Complex 1 group (*p* < 0.01), whereas both liver area and delayed yolk sac absorption area were significantly increased in the Complex 2 group (*p* < 0.01, Figure 4B). In contrast, neither mixture concentration caused a significant change in liver brightness (*p* > 0.05, Figure 4B).

**Figure 5:**
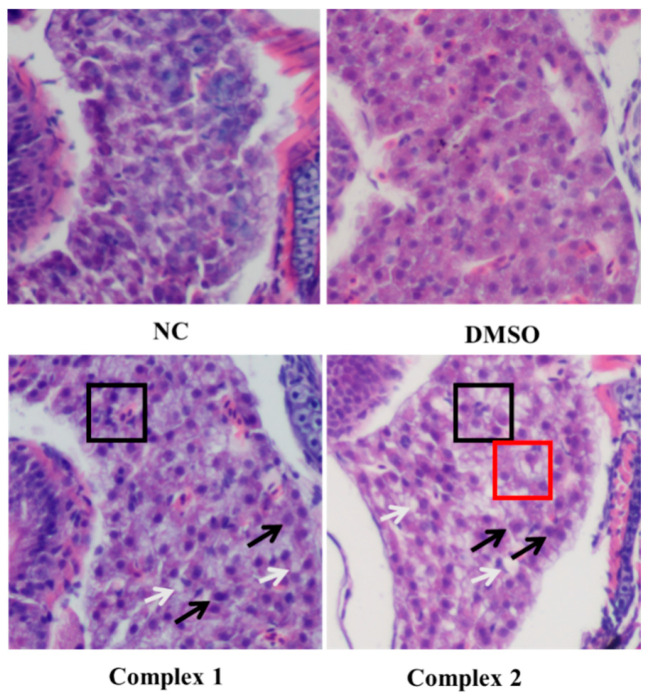
Effects of Maillard reaction product complexes on liver pathology. Black arrows point to nuclear enlargement, and white arrows point to vacuolar degeneration. Red boxes indicate cytoplasmic edema and pale staining, and black boxes indicate necrotic foci.

Histopathological changes in the liver were further evaluated by hematoxylin and eosin (H&E) staining, as shown in Figure 5. In the normal control group and solvent control group (1% DMSO), hepatocytes exhibited abundant cytoplasm, large round nuclei, clear cellular architecture, and regular arrangement, with only a small number of lipid droplets. In the 1/9 MNLC group, a focal area of hepatocyte necrosis was observed, accompanied by enlarged nuclei and vacuolar degeneration. In the 1/3 MNLC group, focal hepatocyte necrosis was observed, together with nuclear enlargement, cytoplasmic edema and degeneration, loose and pale staining, and vacuolar degeneration. These results indicate that hepatotoxicity induced by the MRP mixtures was primarily characterized by increased liver area and delayed yolk sac absorption. Notably, Complex 2 exerted greater hepatotoxicity than Complex 1.

### 3.6. Comparison of Toxicity Between Mixtures and Single Compounds

**Figure 6:**
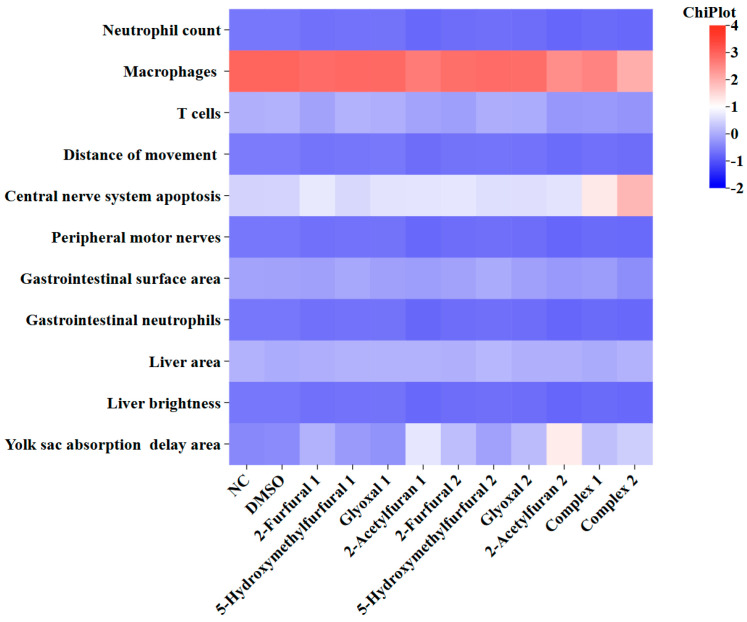
Comparison of the toxicity of Maillard reaction product complexes and single compounds.

Our results demonstrated that the four-compound mixture induced immunotoxic, neurotoxic, gastrointestinal, and hepatotoxic effects. To determine whether interactions among different Maillard reaction products enhanced toxicity, we compared the effects of the four individual compounds with those of the mixture, as shown in Figure 6. Regarding immunotoxicity, the individual compounds and the mixture had similar effects on neutrophil counts. However, compared with single-compound exposure, mixture exposure resulted in a greater reduction in macrophages and T cells, with the most pronounced effect observed in macrophages. Regarding neurotoxicity, mixture exposure at both concentrations produced a greater increase in apoptosis in central nervous system cells than single-compound exposure. In contrast, the mixture did not appear to exert substantially greater gastrointestinal or hepatic toxicity than the individual compounds. In summary, our results suggest that the four Maillard reaction products exert synergistically enhanced toxicity, primarily affecting the immune and nervous systems.

## 4. Discussion

This study focused on the major MRPs detected in infant formula and established two mixture exposure systems consisting of glyoxal, 2-acetylfuran, 2-furfural, and 5-hydroxymethylfurfural. The immunotoxic, neurotoxic, gastrointestinal, and hepatotoxic effects of these mixtures were systematically evaluated in zebrafish. Our results indicate that infant formula contains not a single MRP, but multiple coexisting carbonyl- and furan-derived thermal processing products. Furthermore, combined exposure at levels below or close to the non-lethal dose range of the individual compounds was sufficient to induce clear immune and neurological damage. Moreover, compared with the gastrointestinal tract and liver, the immune and nervous systems appeared to be more sensitive to combined MRP exposure. These findings suggest that safety assessments of thermally processed foods should not rely solely on individual compounds.

In this study, 2-acetylfuran, 2-furfural, and 5-HMF were detected in all three infant formula samples, whereas glyoxal was detected in two samples, indicating that exposure to MRPs in infant formula often occurs as mixed exposure rather than as exposure to isolated compounds. Glyoxal is a reactive dicarbonyl compound and an important precursor of AGEs [9]. 5-HMF, 2-furfural, and 2-acetylfuran are common furan- and carbonyl-containing byproducts of thermal processing and are representative of such compounds in heat-processed foods such as milk powder [4,10]. Both spray drying during infant formula production and subsequent storage promote carbonylation, dehydration, and cleavage reactions, leading to the formation of various low-molecular-weight MRPs. In addition, reactive dicarbonyl compounds in food may continue to participate in AGE formation and protein modification, thereby amplifying potential biological effects. Previous studies have suggested that the major challenge in controlling the Maillard reaction lies not in any single product, but in the complexity of the downstream reaction network, the coexistence of multiple products, and their continuous interconversion.

With regard to immunotoxicity, this study found that both mixture concentrations significantly reduced the fluorescence signals of neutrophils and macrophages, whereas a decrease in T-cell fluorescence was observed only at the higher concentration. Neutrophils and macrophages represent rapid effector cells and phagocytic or inflammatory regulatory cells in the innate immune system, respectively; decreases in their numbers or fluorescence intensity typically indicate impaired host capacity for pathogen clearance, inflammatory initiation, and responses to tissue damage [25,26]. In contrast, T cells belong to the adaptive immune system, and their maturation status and sensitivity thresholds to toxic exposure may differ from those of myeloid cells [27]. This may explain the absence of significant changes in T cells at lower exposure levels. These findings suggest that MRPs may initially disrupt myeloid cell homeostasis and, at higher doses, extend their effects to lymphocytes. Because reactive carbonyl compounds such as glyoxal can induce protein–nucleic acid cross-linking, oxidative stress, and inflammatory dysregulation [28,29], and because 5-HMF and furan compounds have also been reported to induce cytotoxicity and oxidative damage, the decline in innate immune cells under combined exposure is likely associated with the combined effects of carbonyl stress and apoptosis [30]. Notably, Complex 2 caused significantly greater neutrophil damage than Complex 1, whereas no significant difference in macrophage damage was observed between the two doses. This indicates that the dose–response relationship of mixed MRPs varies among immune cell subsets. Neutrophils, which exhibit rapid turnover and high metabolic activity, may be more sensitive to oxidative or carbonyl stress and therefore more likely to show clear dose-dependent effects [31]. Compared with individual compounds, the mixture exerted stronger effects on macrophages and T cells than the single compounds.

The neurotoxicity findings also showed a dose-dependent pattern. Following mixture exposure, apoptosis in central nervous system cells increased significantly, and this effect was further amplified in the high-dose group. Meanwhile, peripheral motor neuron shortening was observed only in the high-dose group. No significant difference in total movement distance was detected during the 20 min dark period, whereas reduced movement speed was observed only during the light phase. Previous studies have shown that light–dark behavior and visuomotor responses in zebrafish larvae can be used to identify developmental neurotoxins; however, these endpoints are highly dependent on the illumination phase, time interval, and stimulus pattern [32,33]. The increased central apoptosis and shortened motor neurons induced by the 1/3 MNLC mixture suggest that this MRP mixture is neurotoxic to developing neurons. Previous studies on 5-HMF, 2-acetylfuran, and 2-furfural have indicated that these thermal processing byproducts can induce varying degrees of neurodevelopmental toxicity in zebrafish [22,34]. Oxidative stress is considered a common mechanism underlying multiple forms of chemical neurotoxicity. Because glyoxal is also a precursor of AGE formation, it may amplify neuronal damage caused by other components through protein cross-linking, mitochondrial stress, and reactive oxygen species accumulation [35,36].

The gastrointestinal toxicity results showed that the low-dose mixture did not alter gastrointestinal area, whereas the high-dose mixture significantly reduced it. However, the number of gastrointestinal neutrophils per unit area remained unchanged. In zebrafish larvae, gastrointestinal area is commonly used as a simple indicator of intestinal growth, morphological integrity, and developmental progress; a reduction in this parameter typically suggests suppressed epithelial proliferation, developmental delay, or local tissue damage [37]. In contrast, intestinal neutrophil number is more indicative of mucosal immune activation [24]. Previous zebrafish studies have shown that intestinal neutrophil levels are maintained in a microbiota-dependent steady state [38]. When host–microbe signaling is altered, intestinal neutrophil levels may either increase or decrease [39]. In the present study, the mixture did not alter intestinal neutrophil levels but reduced intestinal area, suggesting that the early effects of the MRP mixture on the gastrointestinal tract primarily involve developmental toxicity rather than marked local inflammatory cell recruitment.

The liver is a critical organ for host metabolism and detoxification. Liver area, liver brightness, and delayed yolk sac resorption area are common morphological endpoints used to evaluate hepatotoxicity in zebrafish larvae [40]. Among these endpoints, delayed yolk sac resorption typically reflects impaired lipid mobilization and energy metabolism and is often associated with abnormal liver development, reduced metabolic capacity, or an increased detoxification burden [41]. Changes in liver area may reflect altered organ growth, swelling, or atrophy. H&E staining can reveal parenchymal damage such as cellular swelling, vacuolization, and necrosis. Previous studies have indicated that liver damage induced by drugs or environmental toxicants in zebrafish is often accompanied by abnormal liver morphology and delayed yolk sac resorption [42]. In this study, the Complex 1 group showed increased delayed yolk sac absorption, whereas the Complex 2 group additionally exhibited increased liver area, histological necrosis, and vacuolization, indicating that the MRP mixture induced typical hepatotoxicity.

By comparing the mixture with its individual components, we found evidence of synergistic effects among the four typical MRPs. However, this synergy appeared to be organ-specific, being most evident in the immune and nervous systems, while not consistently exceeding the effects of individual components in the gastrointestinal tract and liver. This finding suggests that, in food safety assessment, the toxicity of mixed exposure does not manifest as a uniform increase across all endpoints, but rather as selective amplification of specific highly sensitive biological processes. Compared with previous single-compound exposure studies, the mixture produced a broader toxicity profile. Glyoxal alone caused immune, gastrointestinal, and hepatic injury at LC10, with limited neurobehavioral effects, whereas 2-acetylfuran showed immune and hepatic sensitivity and neurological or gastrointestinal toxicity mainly near MNLC or LC10. For 2-furfural and 5-HMF, responses were also organ- and concentration-dependent, with 2-furfural showing stronger neurotoxicity and 5-HMF showing limited GI effects. In contrast, Complex 1 already induced immune suppression and CNS apoptosis, whereas Complex 2 additionally impaired T cells, motor neurons, GI development, and liver morphology, indicating concentration-dependent enhancement under combined exposure. The immune and nervous systems are both characterized by high energy demand, rapid responsiveness, and finely tuned regulation. In addition, both systems are highly sensitive to oxidative stress and protein modification. Consequently, they may be more susceptible to enhanced toxicity under combined exposure.

This study also has two limitations. First, the current endpoints were primarily morphological and fluorescence-based; future studies should incorporate molecular markers related to oxidative stress, inflammation, and apoptotic pathways to validate the mechanistic basis of the observed synergistic effects. Second, Secondly, infant formula is composed of complex ingredients, and the actual risk of Maillard reaction product exposure may also be influenced by matrix effects. Future research should focus on the impact of matrix effects on the risk of Maillard reaction product exposure from food. Nevertheless, this study demonstrates that multiple MRPs commonly found in infant formula can, when present in combination, cause greater damage to the host immune and nervous systems even at low doses, thereby providing a theoretical basis for improving the control of thermal processing and storage conditions in infant formula production.

## 5. Conclusions

Based on typical Maillard reaction products (MRPs) detected in infant formula, this study established a mixed-exposure system comprising glyoxal, 2-acetylfuran, 2-furfural, and 5-hydroxymethylfurfural. Using a zebrafish model, the developmental and multi-organ toxicity of this mixture was systematically evaluated. The results indicate that MRPs in infant formula exhibit clear co-exposure characteristics and that their toxic effects cannot be fully predicted from the additive effects of individual compounds. Compared with single-compound exposure, combined exposure induced more pronounced immunosuppression and neurotoxicity at lower doses, as evidenced by reduced numbers of neutrophils, macrophages, and T cells, increased neuronal apoptosis, and impaired motor neuron development. In addition, the high-dose mixture caused gastrointestinal developmental abnormalities, liver morphological changes, delayed yolk sac absorption, and more severe histopathological damage. In summary, this study provides a theoretical basis for improving the control of Maillard reaction product toxicity in foods.

## Figures and Tables

**Table 1:** Identification of 12 Maillard reaction products in infant formula (n = 3).

Standards	Retention Time	IF1	IF2	IF3
glyoxal	2.110	No	Yes	Yes
2,3-Butanedione	2.744	No	No	No
2-acetylfuran	3.270	Yes	Yes	Yes
L-Lysine hydrochloride	4.327	No	No	No
Diacetyl	5.262	No	No	No
Nα,Nα-di(carboxymethyl)-L-lysine	5.393	No	No	No
NƐ-acetyl-L-lysine	6.272	No	No	No
Acetone alcohol	6.843	No	No	No
2-furfural	7.737	Yes	Yes	Yes
5-hydroxymethylfurfural	9.668	Yes	Yes	Yes
Schiff base	13.630	No	No	No
Fructose-lysine	14.372	No	No	No

**Table 2:** Analysis of zebrafish movement speed under light and dark conditions.

Grouping	1–5 Min (Light)	6–10 Min (Dark)	11–15 Min (Light)	16–20 Min (Dark)
NC	1.980 ± 0.067	0.672 ± 0.075	2.000 ± 0.020	0.565 ± 0.038
DMSO	1.900 ± 0.052	0.466 ± 0.106	1.720 ± 0.181	0.531 ± 0.082
Complex 1	1.540 ± 0.020 ***	0.522 ± 0.085	1.24 ± 0.028 *	0.736 ± 0.048
Complex 2	1.630 ± 0.024 **	0.395 ± 0.096	1.570 ± 0.086	0.339 ± 0.067

* *p* < 0.05, ** *p* < 0.01, *** *p* < 0.001. n = 10. NC: Normal control group, DMSO: solvent control group, Complex 1 is a composition of four Maillard reaction products at a concentration of 1/9 MNLC, and Complex 2 is a composition of four Maillard reaction products at a concentration of 1/3 MNLC.

## Data Availability

The original contributions presented in this study are included in the article. Further inquiries can be directed to the corresponding author.
